# Epigallocatechin gallate (EGCG) suppresses epithelial-Mesenchymal transition (EMT) and invasion in anaplastic thyroid carcinoma cells through blocking of TGF-β1/Smad signaling pathways

**DOI:** 10.1080/21655979.2019.1632669

**Published:** 2019-07-16

**Authors:** Tingting Li, Ning Zhao, Jie Lu, Qingli Zhu, Xinfeng Liu, Fengyun Hao, Xuelong Jiao

**Affiliations:** a Department of Endocrinology, Linyi Central Hospital, Linyi, Yishui, Shandong, China; b Department of Thyroid Surgery, The Affiliated Hospital of Qingdao University, Qingdao, Shandong, China; c Department of Nuclear Medicine, The Affiliated Hospital of Qingdao University, Qingdao, Shandong, China; d Department of Pathology, The Affiliated Hospital of Qingdao University, Qingdao, Shandong, China; e Department of General surgery, The Affiliated Hospital of Qingdao University, Qingdao, Shandong, China

**Keywords:** Anaplastic thyroid carcinoma (ATC), transforming growth factor, Smad, epithelial-to-mesenchymal transition

## Abstract

Transforming growth factor (TGF)-β1 plays a crucial role in the epithelial-to-mesenchymal transition (EMT) in many cancer types and in thyroid cancers. Epigallocatechin-3-gallate (EGCG), the most important ingredient in the green tea, has been reported to possess antioxidant and anticancer activities. However, the cellular and molecular mechanisms explaining its action have not been completely understood. In this study, we found that EGCG significantly suppresses EMT, invasion and migration in anaplastic thyroid carcinoma (ATC) 8505C cells *in vitro* by regulating the TGF-β/Smad signaling pathways. EGCG significantly inhibited TGF-β1-induced expression of EMT markers (E-cadherin reduction and vimentin induction) in 8505C cells *in vitro*. Treatment with EGCG completely blocked the phosphorylation of Smad2/3, translocation of Smad4. Taken together, these results suggest that EGCG suppresses EMT and invasion and migration by blocking TGFβ/Smad signaling pathways.

## Graphical Abstract

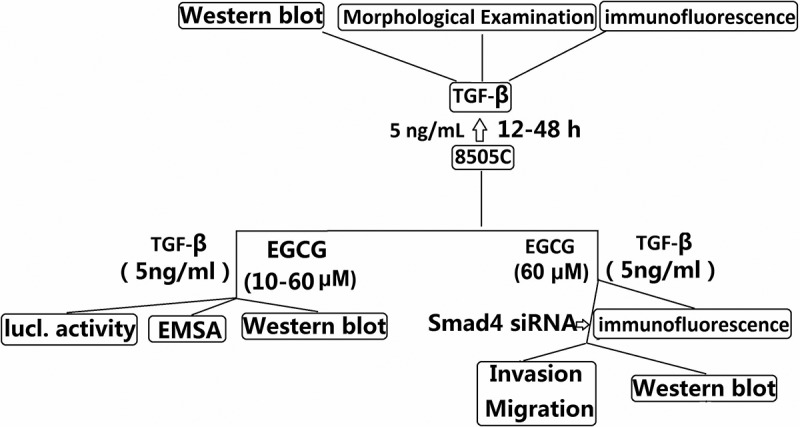

## Introduction

Transforming growth factor β (TGF-β), a ubiquitously expressed cytokine, may act as both a tumor suppressor and a potent stimulator of tumor progression, local invasion, and metastasis [1]. TGF-β signaling regulates various biological processes including cell growth, differentiation, angiogenesis, apoptosis, and extracellular matrix remodeling [2]. Alterations in TGF-β signaling are linked to a variety of human diseases including cancer, inflammation, and tissue fibrosis [3,4]. Ligand binding to TGF-β receptors initiates Smad2/3/4 complex formation and translocation to the nucleus (Smad pathway) [2]. Once inside the nucleus, the complex binds to sites within gene promoter regions termed SMAD-binding elements (SBEs) in order to control gene expression [5].

A phenomenon known as epithelial to mesenchyme transition (EMT) has been implicated as having a role in tumor invasion/migration and metastasis [6]. Loss of E-cadherin expression is a hallmark of the EMT process, which is probably required for enhanced tumor-cell motility. TGF-β is a major factor enhancing tumor progression, epithelial-mesenchymal transition (EMT), and invasiveness and metastatic capacity [7]. TGF-β can induce typical EMT in culture lung cancer cells [8,9] and thyroid cancer cells [10]. Pirozzi et al used two epithelial cell lines which acquired a fibroblast-like appearance when treated by TGF-β1. By inhibiting TGF-β1, vimentin and CD90 were downregulated and cytokeratin, E-cadherin, and CD326 were upregulated. TGF-β1 also upregulated Slug, Twist, and β-catenin, thus confirming EMT [11].

Clinically evident indicated that 50% of ATC patients have had distant metastases at the time of clinical diagnosis, as to the ATC patients who have not had distant metastases. They will also be classified as stage IV by the American Joint Committee on Cancer. Although metastasis is common in ATC, not much is known about specific molecular mechanisms. Our previous study showed targeting TGF-β1 expression in ATC 8505C cells caused a 70% decrease in migration and a 78% decrease in invasion [12,13]. In papillary thyroid carcinoma and ATC cells, EMT was induced by TGF-β1 and increased tumor invasiveness in PTCs [14,15]. These data indicated that targeting TGF-β1 expression could reversed EMT and inhibited ATC cells invasion.

Several small-molecule inhibitors (SMI) have been developed to block the TGF-β signaling pathway with the intention to reduce tumor growth [16,17]. Green tea is a beverage that is widely consumed worldwide and is believed to exert effects on different diseases, including cancer. The major components of green tea are catechins, a family of polyphenols [18]. Among them, epigallocatechin-gallate (EGCG) is the most abundant and biologically active. EGCG is widely studied for its anti-cancer properties. EGCG is effective in vivo at micromolar concentrations, suggesting that its action is mediated by interaction with specific targets that are involved in the regulation of crucial steps of cell proliferation, survival, and metastatic spread [19]. Chang et al has recently reported that EGCG dose-dependently inhibited thrombin-induced TGF-β1 activation [20]. Wang et al has reported that EGCG prevented TGF-β1 mediated EMT and Smad 2 and Smad 3 phosphorylation in a dose dependent manner in NRK-52E cells [21]. EGCG also inhibits TGF-β1-mediated EMT by suppressing the acetylation of Smad2 and Smad3 in human lung cancer cells [22]. De Amicis et al slao reported that the inhibitory role of EGCG on thyroid cancer cell proliferation and motility with concomitant loss of epithelial-to-mesenchymal cell transition markers [23]. However, these studies are insufficient to demonstrate that EGCG can prevent EMT in vitro. Therefore, we further evaluated the molecular mechanism underlying the anti-EMT activities of EGCG by observing its effect in vitro. We demonstrated that EGCG suppressed EMT and invasion by affecting multiple TGF-β1-mediated molecular mediators involved in ATC cells.

## Materials and methods

### Cell cultures

The human ATC line 8505C was obtained from German Collection of Microorganisms and Cell Culture (DSMZ, Braunschweig, Germany) and kept in our laboratory. The 8505C cell line was also authenticated using short tandem repeat (STR) analysis as described in 2012 in ANSI Standard (ANSI/ATCC ASN-0002–2011 Authentication of Human Cell Lines: Standardization of STR Profiling) by the ATCC Standards Development Organization (SDO) and negative for mycoplasma contamination, carried out by Guangdong Hybribio Biotech Ltd,. The 8505C cells were routinely cultured in DMEM/F12 (Thermo Fisher scientific, CA, USA) medium, supplemented with 10% fetal bovine serum (Thermo Fisher scientific) and penicillin (100 U/ml) and streptomycin (100 μg/ml) (Thermo Fisher scientific), and maintained in a humidified 5% CO2 atmosphere at 37°C.

### Reagents

Human TGF-β1 and Smad4 was obtained from Sigma-Aldrich, Castle Hill, NSW, Australia; Signal inhibitors Smad4 siRNA and control siRNA were obtained from Calbiochem (Calbiochem, Cambridge, MA, USA). Epigallocatechin gallate (EGCG) (purity >98%), which was purchased from Chengdu Herbpurify Co., Ltd (Chengdu, Sichuan, China), was dissolved in PBS with 2% dimethyl sulfoxide (DMSO; Sigma‐Aldrich, St. Louis, MO, USA). Mouse monoclonal antibodies for cytokeratin, vimentin, F-actin, E-cadherin and a goat polyclonal antibody for phosphorylated (p)-Smad2/3/4 were used as primary antibodies were from Santa Cruz Biotechnology, Inc., Shanghai, China. All chemicals were obtained from Sigma-Aldrich unless otherwise indicated.

### RNA interference

siRNA targeting Smad4 and a negative control siRNA were synthesized by GenePharma (Shanghai, China). 8505C cells (2 × 10^5^) were seeded into 6-well plates and treated with 100 nM siRNA using Lipofectamine™ 2000 (Invitrogen, CA, USA) according to the manufacturer’s instructions. The cells were used for further experiments after 24 h of transfection.

### Cell treatments

After 24 h of serum starvation, 8505C cells were maintained in growth media supplemented with 5 ng/mL TGF-β1 (dissolved in 10 mM citric acid, pH = 3.0) for 12–48 h to induce EMT. In the majority of experiments, cells were maintained in media supplemented with TGF-β1 with or without EGCG (10, 40, 60 μM) for 48 h to investigate the effects of EGCG on EMT in TGF-β1 treated 8505C cells. To investigate pathways possibly involved in the effect of EGCG on EMT, the cells were transfected into 100 nM Smad4 siRNA or control siRNA 24h before treatment with TGF-β1 with or without EGCG. Cells were harvested at the indicated time points for protein extraction and western blotting and EMSA assay.

### Boyden chamber transwell assay

8505C cells or 8505C cells transfected with Smad4 siRNA were maintained in growth media supplemented with 5 ng/mL TGF-β with or without EGCG (60 μM) for 24 h. Cells were plated on top of a thick layer of Matrigel in transwell chambers (BD Biosciences). After culturing for 18 hours, non-invasive cells on the upper surface of filters were removed completely. Invasive cells adhered to the lower surface of filter were rinsed with phosphate-buffered saline (PBS), fixed with methanol, stained with 0.05% crystal violet and counted.

### Wound healing assay

Cells were seeded and allowed to reach 70%–80% confluence, then starved for 36 hours. The cell monolayers were then wounded with a sterile plastic tip and cultured in serum-free medium. Cell migration was monitored 48 hours using microscopy (Nikon, Tokyo, Japan).

### Western blot assay

Cells were lysed in RIPA and protein concentrations were determined using the BCA protein assay kit (P0010, Beyotime Biotechnology, Shanghai, China). Equal amounts of proteins (40 μg) were resolved by 10% SDS-PAGE and the proteins were transferred to Hybond ECL membranes (Amersham, Buckinghamshire, UK). The membranes were incubated with primary antibodies at 4°C overnight. The primary antibodies used included anti- vimentin, Smad2/3/4, phosphorylated Smad2/3/4, E-Cadherin, cytokeratin, F-actin, Snail/Slug, Twist, Zeb1 and **β**-actin. After washing with TBST, the membranes were probed with HRP-labeled secondary antibodies. The membranes were visualized using an enhanced chemiluminescence system (Kodak, Rochester, NY, USA).The densitometric data of Western blots of the three independent experiments were combined.

### DNA transfection and luciferase assay

Reporter gene activity was evaluated by cell-based analysis methods for assaying Smad3/4 activity. To measure TGF-β1 signaling, 8505C cells were maintained in media supplemented with TGF-β1 with or without EGCG (10, 40, 60 μM) for 48 h. Then the cells were cotransfected with 100 ng of (CAGA)12-Luc reporter and 0.2 ug of the Renilla reporter plasmid for 6 h using Lipofectamine reagent (Invitrogen, Carlsbad, CA, USA) according to the manufacturer’s protocol. After transfection, cells were pretreated with MEL for 1 h and then stimulated with TGF-β1 for 24 h. Luciferase and Renilla activities were determined by following the manufacturer’s protocol.

### DNA binding activity of SBE and Smad4 antibody

DIG Gel Shift Kit (Roche, Mannheim, Germany) was performed to detect Smad binding element (SBE) DNA-binding and Smad4 antibody-binding activity, with the instructions of manufacturer. The binding activity of Smad 3/4 in nuclear extract of 8505C was confirmed by EMSA or supershift assay with a DIG-labeled oligonucleotide probe forward 5’-AGTATGTCTAGACTGA-3’; SBE antisense 5’-TCAGTCTAGACATACT-3’ and Smad4 antibody. EMSA was performed by incubating 10 ug of nuclear extract in a 9 uL binding reaction mixture at 37 °C for 10 min. The binding reaction mixture for the super shift assay containing 1 uL of the non-diluted antibody of Smad4 was added to 1 uL of DIG-labeled double-stranded oligonucleotide and was incubated at 37 °C for 20 min, followed by the addition of 1 uL of the gel loading 10ul buffer at room temperature. The DNA-protein complexes were separated by electrophoresis in 6% non-denaturing polyacrylamide gels using 0.25 ul Tris-borate-EDTA as a running buffer. After electrophoresis, the gels were transferred to nylon membranes and detected chemiluminescent. The luminescent signals were analyzed using an ImageQuant LAS 4000 Scanner of GE Healthcare.

### Morphological examination and immunofluorescence studies

8505C cells were treated with 5 ng/mL TGF-β1 for 48 h. Morphological changes in the 8505C cells were observed under a Leica DMIL LED phase contrast microscope with an attached EC3 camera (Leica, Germany). The photographs were taken at 200 x magnification. Immunofluorescence staining was used to detect E-cadherin, cytokeratin, vimentin, and F-actin in the 8505C cells as the manufacture’s instruction. The fluorescent images were obtained by confocal laser scanning microscope (Zeiss LSM 510, Oberkochen, Germany).

After 8505 cells were maintained in media supplemented with 5 ng/mL TGF-β1 with or without 60 μM EGCG for 48 h. 8505C cells were washed with phosphate-buffered saline (PBS) and fixed in 4% paraformaldehyde. After

permeabilization with 0.5% Triton X-100 and blocking in 5% bovine serum albumin (BSA), the cells were incubated

with primary E-cadherin or vimentin antibodies overnight at 4°C then incubated with fluorescence-conjugated secondary antibodies at room temperature for 1 h. The nuclei were stained with 4’, 6-diamidino-2-phenylindole (DAPI) for 5 min. Images were pseudocolored using a Zeiss Instruments confocal microscope.

### Statistical analysis

All data are presented as the mean value ± SD. Differences between individual groups were analyzed by t-tests. P values less than 0.05 (two-sided) were considered statistically significant. Statistical analyses were performed using the SPSS 11.5 software (SPSS Inc).

## Results

### Morphologic characteristics in 8505C cells in response to TGF-β1

**Figure 1.**
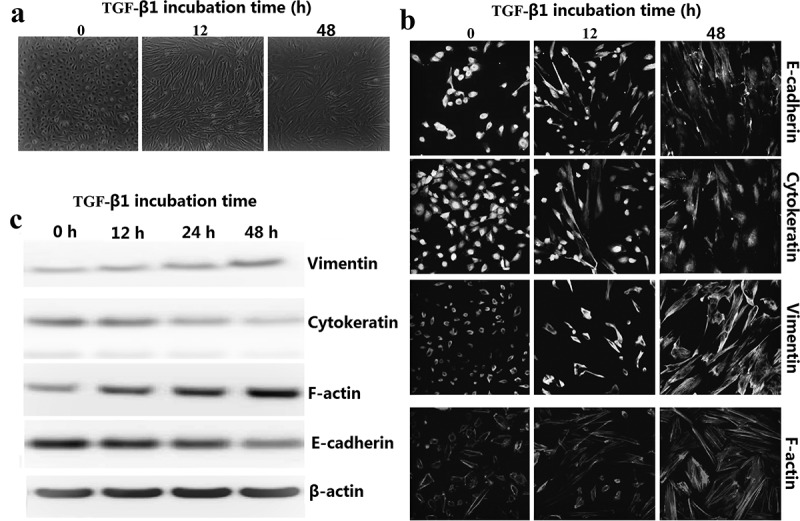
TGF-β1-stimulated EMT in 8505C cells. (a) Time effect of TGF-β1 on EMT were examined by morphologic changes in 8505C, 200× magnification. (b) Immunofluorescence stains: Cultured 8505C cells after TGF-β1 treatment were stained with monoclonal antibodies against E-cadherin, cytokeratin, vimentin and F-actin. (c) Expression EMT markers in TGF-β1–stimulated 8505C cells was detected by western blot assay.

TGF-β1-induced EMT was performed using the cultured lung cancer 8505C cells according to a method described previously [24]. In response to TGF-β1, 8505C acquired a spindle-like mesenchymal morphology, which could be detected after 12 h and became prominent after 48 h (Figure 1(a)). Immunofluorescence microscopy demonstrated the loss of E-cadherin, cytokeratin with the replacement by vimentin, and the stress fiber reorganization by F-actin (Figure 1(b)). We determined the expression of EMT markers in TGF-β1-stimulated 8505C cells by western blot assay. TGF-β1 treatment stimulated vimentin and F-actin expression time-dependently in 8505C cells. In addition, downregulation of E-cadherin and cytokeratin occurred during TGF-β1 treatment in a time-dependent manner (Figure 1(c)).

### Effects of EGCG on TGF-β1-induced EMT in vitro

**Figure 2.**
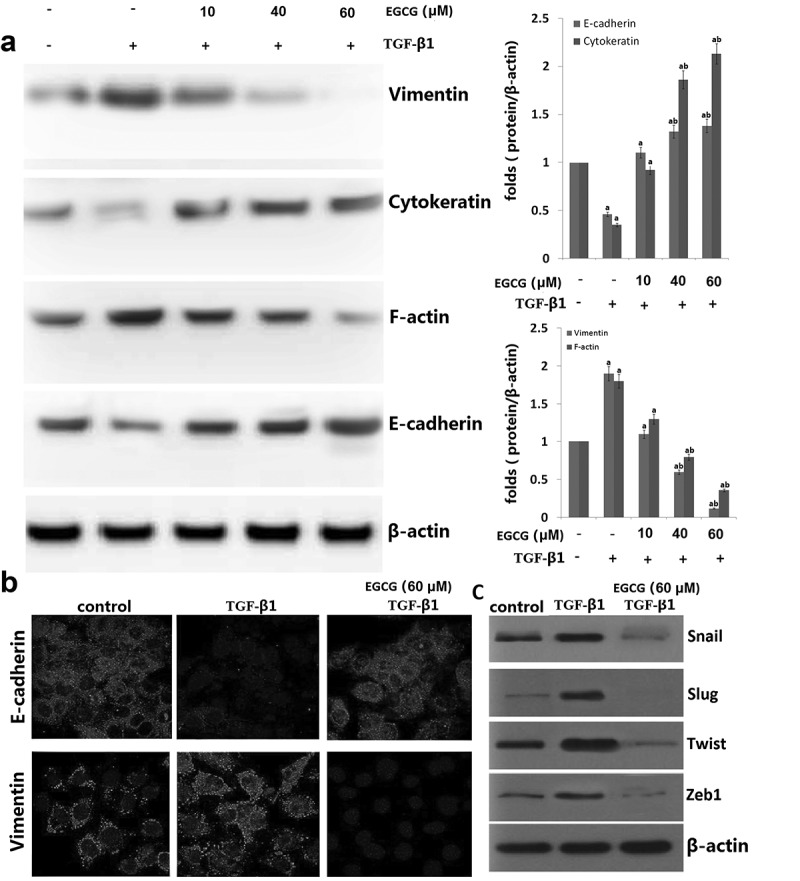
Effects of EGCG on TGF-β1-induced epithelial-to-mesenchymal transition (EMT) in vitro. 8505C cells were pretreated for 1 h with EGCG, followed by incubation with TGF-β1 for 48 h. (a) Expression of EMT markers in TGF-β1-stimulated 8505C cells was detected by western blot assay. (b) Immunofluorescence double staining for E-cadherin (red) and vimentin (red) in TGF-β1-stimulated 8505C after treatment of EGCG. Cells was counterstained with Hoechst 33342 (blue). Magnifications ×200; (c) Expression of the E-cadherin transcription repressors in TGF-β1-stimulated 8505C cells was detected by western blot assay. The data are representative of three similar experiments and quantified as mean values ± S.E, vs control, ^a^P<0.05; vs TGF-β1,^b^P<0.05.

To investigate the effect of EGCG in TGF-β1-EMT progression, 8505C cells was pretreated with EGCG for 1 h and then stimulated with TGF-β1 for 48 h (Figure 2(a)). EGCG treatment resulted in cellular resistance to expression of EMT markers (epithelial marker: E-cadherin and cytokeratin, mesenchymal marker: F-actin and vimentin) in a dose-dependent manner. We also carried out immunofluorescence staining to examine the expression of E-cadherin and vimentin in 8505C cells. As shown in Figure 2(b), EGCG abrogated downregulation of E-cadherin and upregulation of vimentin expression. Consistent with these results, TGF-β1 treatment reduced membrane-associated expression of E-cadherin, with a loss of expression at cell borders and concomitant increases in vimentin expression in a fibril-associated pattern. 8505C cells concurrently treated with EGCG and TGF-β1 maintained high levels of localized expression of E-cadherin; they also showed no increase in the levels of mesenchymal markers.

Candidate E-cadherin transcription repressors are members of the Snail, Twist and ZEB families. Therefore, we determined whether treatment with EGCG impaired the endogenous expression of Snail1/2, Zeb1 and Twist. As shown in Figure 2(d), EGCG significantly attenuated the expression of Snail1/2, Zeb1 and Twist protein in TGF-β1-stimulated 8505C cells. These results demonstrate that EGCG plays an important role in suppressing TGF-β1-induced EMT in vitro.

### EGCG prevents EMT via TGF-β/Smad signal transduction pathway

**Figure 3.**
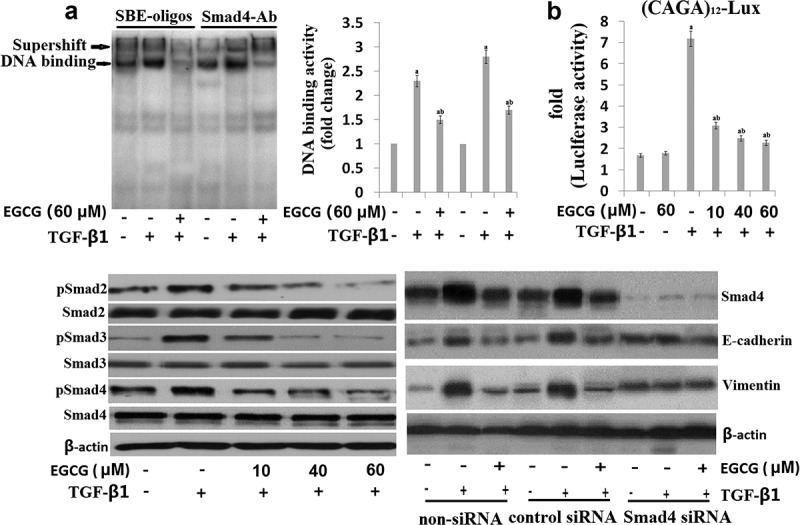
EGCG antagonizes the TGF-β1-stimulated Smad signal pathway in vitro. Cells were pretreated for 1 h with EGCG, followed by incubation with TGF-β1 for 24 h. (a) Nuclear extracts were subjected to SBE DNA binding and Smad4 antibody (Ab) assay by EMSA supershift assay; (b) EGCG inhibits TGF-β1-dependent transcriptional activity of the CAGAx12-Luc reporter in a dose-dependent manner; (c) Immunoblot of the effect of EGCG on the TGF-β1-stimulated pSmad2, pSamd3 and Smad4; (d) 8505C was transfected with control (Con) or specific Smad4 siRNA and then treated with TGF-β1 for 24 h or 48 h. The quantitative ratios are shown as relative optical densities of bands that are normalized to the expression of β-actin. The data are representative of three similar experiments and quantified as mean values ± S.E. ^a^p < 0.05 versus normal control, ^ab^p < 0.05 versus TGF-β1 treatment.

In TGF-β1-mediated EMT, the phosphorylated Smad3/4 complex activates the transcription of TGF-β1-mediated genes through interactions with other DNA-binding transcription factors. To determine whether EGCG antagonizes the effects of TGF-β1 by interfering with Smad3/4 binding to DNA, we performed an EMSA and supershift assay using a SBE-specific sequence and Smad4 Ab complexes. SBE-DNA and Smad4-Ab complexes were strongly increased by TGF-β1, and this increase was inhibited by EGCG (Figure 3(a)). The inhibitory effect of EGCG on TGF-β1-dependent gene transcription of the (CAGA) 12-Lux reporter was observed in nature (Figure 3(b)). Moreover, EGCG abrogated the TGF-β1-mediated phosphorylation of Smad2/3 in a dose-dependent manner (Figure 3(c)). Upon phosphorylation, Smad2/3 forms a complex with the co-mediator Smad4 and subsequently translocate into the nucleus. Within the nucleus, the Smad2/3/4 complex regulates the transcription of target genes responsible for EMT. We examined the role of TGF-β1/Smad in EMT regulation by pretreating 8505C with Smad4 siRNA. After transfection with Smad4 siRNA for 24 h, the expression of Smad4 was declined in TGF-β1-stimulated 8505C (Figure 3(d)). In addition, EGCG treatment alone or Smad4 siRNA transfection alone markedly increased E-cadherin expression and reduced vimentin expression in TGF-β1-stimulated 8505C. However, non-siRNA alone or transfection with control siRNA alone did not affect Smad4 accumulation in TGF-β1-stimulated 8505C cells (Figure 3(d)). Taken together, these results suggest that EGCG aEGCGiorates TGF-β1-mediated EMT by blocking the TGF-β1/canonical Smad pathway.

### EGCG inhibits invasion and migration of 8505C cells via TGF-β/Smad signal transduction pathway

**Figure 4.**
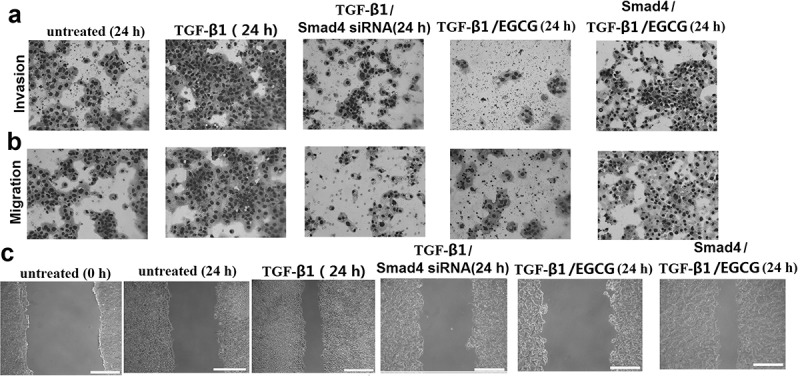
EGCG inhibits invasion and migration of 8505C cells via TGF-β/Smad4 Signal transduction pathway. 8505C cells were maintained in growth media supplemented with 5 ng/mL TGF-β with or without EGCG (60 μM) for 24 h. Or the 8505C cells were transfected with Smad4 siRNA or treated with Smad4 24 h before the TGF-β with or without EGCG treatment. (a) Boyden chamber assay performed to evaluate cell invasion; (b) Boyden chamber assay performed to evaluate migration; (c) Cell migration determined by the wound-healing assay. Magnification ×100 and scale bars = 100 μm.

It has shown that EGCG prevents EMT via TGF-β/Smad Signal transduction pathway. To further demonstrate that TGF-β/Smad signaling pathway is essential for the effects of EGCG, 8505C cells were maintained in growth media supplemented with 5 ng/mL TGF-β with or without EGCG (60 μM) for 24 h. Upon TGF-β1 stimulation, EGCG-induced migratory and invasive abilities in the 8505C cells was restored (Figure 4(a–c)). Furthermore, in the presence of TGF-β1, 8505C cells transfected with Smad4 siRNA showed decreased migratory and invasive abilities in the 8505C cells compared to the presence of TGF-β1 alone (Figure 4(a-c)). In contrast, in the presence of TGF-β1 and EGCG, 8505C cells transfected with Smad4 showed decreased migratory and invasive abilities in the 8505C cells compared to the presence of TGF-β1 and EGCG alone (Figure 4(a–c)). These results indicate that EGCG is effective in the prevention of TGF-β1-induced ATC cell invasion and migration.

## Discussion

This study is the first to demonstrate that EGCG inhibits TGF-β1-induced epithelial to mesenchymal transition (EMT) in 8505C cells. EGCG significantly reduced the expression of mesenchymal markers, increased the expression of epithelial markers after TGF-β1 stimulation, and reverted the TGF-β1-induced morphological changes in 8505C cells. We also found that the inhibitory effects of EGCG on TGF-β1-induced EMT may be related to inhibition of the TGF-β1-mediated signaling pathways Smad2/3/4.

Clinical studies have revealed that EMT is closely related to tumor metastasis and poor prognosis and is considered to be the central mechanism responsible for metastasis in multiple cancer, including ATC [25–27]. Numerous studies have reported EMT as a response to TGF-β1 treatment *in vitro* in ATC cells [15]. The mechanism of TGF-β1-induced EMT is complicated because TGF-β1 signals are transmitted through multiple pathways, including the Smad, MAPK and PI3K pathways. The TGF-β1 signal itself is predominantly transduced by the Smad proteins, including Smad2, Smad3 and Smad4 [28]. The major polyphenolic component of dried green tea extracts is epigallocatechin-gallate (EGCG) EGCG is the most abundant and biologically active catechin from green tea, accounting for at least 50% of the total catechin content in green tea leaves. Several in vitro, in vivo, and clinical studies have shown multiple EGCG anticancer actions. Among them there are anti-proliferative, pro-apoptotic, anti-angiogenic, and anti-invasive functions [29]. Accumulating data suggests that EGCG inhibited the epithelial-mesenchymal transition (EMT), which is one of the primary and early pathways involved in cancer development and metastasis [30,31]. Binding of TGF-β1 to its receptors results in the phosphorylation of Smad proteins, causing translocation of the Smad complex into the nucleus where it regulates the expression of target genes. Although both Smad2 and Smad3 are important for the regulation of TGF-β1 signals, the mechanisms of action and the functions of these two Smads are different. In human lung cancer cells, EGCG inhibits TGF-β1-mediated EMT by suppressing the acetylation of Smad2 and Smad3 [22,32]. In our study, we found that EGCG potently attenuated markers for EMT in vitro in 8505C cells. EGCG potently attenuated Smad3/4 complex nuclear translocation and TGF-β1-dependent transcription of the (CAGA)12-Lux reporter in vitro. Also, EGCG significantly inhibited phosphorylation of Smad2/3 and repressed the expression of transcription factors Snail1/2, Zeb1 and Twist, and up-regulated the expression of E-cadherin.

Metastasis is the primary cause of mortality in most cancer patients [33]. Thus, to understand the molecular

mechanisms of metastasis is one of the most important issues in cancer research. Epithelial-mesenchymal transition (EMT), which enables epithelial cells to acquire invasive mesenchymal phenotype, is attracting increasing attention as an important mechanism for the initial step of metastasis [34,35]. Given the critical role of EMT in metastatic tumor formation, inhibition of EMT can be an important therapeutic strategy to inhibit tumor metastasis [36,37]. In our study, we found that EGCG could promote 8505C cell invasion and metastasis *in vitro* through inactivating EMT process. This study thereby demonstrates, for the first time, that EGCG can inhibit EMT and invasion through TGF-β1 to inactivate smad4 in 8505C cells.

In conclusion, our data demonstrated that EGCG inhibits TGF-β1/Smad signaling, and suppresses EMT progression and invasion/migration *in vitro* in 8505C cells. However, the effect of EGCG *in vivo* need further investigation.

## Highlights

- EGCG inhibits **EMT process**, invasion and migration in 8505C cells in vitro
- EGCG inhibits TGF-β1-induced expression of EMT markers
- EGCG blocked TGF-β1-dependent phosphorylation of Smad2/3 and translocation of Smad4
